# Left Atrial Volume Index Predicts Atrial Fibrillation Recurrence after Catheter Ablation Only in Obese Patients—Brief Report

**DOI:** 10.3390/diagnostics14141570

**Published:** 2024-07-19

**Authors:** Franjo Husam Naji, Jan Alatic, Igor Balevski, David Suran

**Affiliations:** 1 University Clinical Center, 2000 Maribor, Slovenia; 2 Faculty of Medicine, University of Maribor, 2000 Maribor, Slovenia

**Keywords:** atrial fibrillation, catheter ablation, left atrial volume index, body mass index

## Abstract

Background: It has been shown that obesity and a higher body mass index (BMI) are associated with a higher recurrence rate of atrial fibrillation (AF) after successful catheter ablation (CA). The same has been proven for the left atrial volume index (LAVI). It has also been shown that there is a correlation between LAVI and BMI. However, whether the LAVI’s prognostic impact on AF recurrence is BMI-independent remains unclear. Methods: We prospectively included 62 patients with paroxysmal AF who were referred to our institution for CA. All patients underwent radiofrequency CA with standard pulmonary veins isolation. Transthoracic 2-D echocardiography was performed one day after CA to obtain standard measures of cardiac function and morphology. Recurrence was defined as documented AF within 6 months of the follow-up period. Patients were also instructed to visit our outpatient clinic earlier in case of symptoms suggesting AF recurrence. Results: We observed AF recurrence in 27% of patients after 6 months. The mean BMI in our cohort was 29.65 ± 5.08 kg/cm^2^ and the mean LAVI was 38.04 ± 11.38 mL/m^2^. We further divided patients into two groups according to BMI. Even though the LAVI was similar in both groups, we found it to be a significant predictor of AF recurrence only in obese patients (BMI ≥ 30) and not in the non-obese group (BMI < 30). There was also no significant difference in AF recurrence between both cohorts. The significance of the LAVI as an AF recurrence predictor in the obesity group was also confirmed in a multivariate model. Conclusions: According to our results, the LAVI tends to be a significant predictor of AF recurrence after successful catheter ablation in obese patients, but not in normal-weight or overweight patients. This would suggest different mechanisms of AF in non-obese patients in comparison to obese patients. Further studies are needed in this regard.

## 1. Introduction

Atrial fibrillation (AF) is a chronic degenerative disorder of pandemic proportions. It was stated that it correlates with increasing age, as well as with common cardiovascular risk factors such hypertension, diabetes, hyperlipidaemia, and obesity [1]. Studies have been conducted to further enlighten the role of body mass index (BMI), body surface area (BSA), and height and weight in the pathophysiology of atrial remodelling and the subsequent incidence and prevalence of AF [2,3]. Regarding the left atrial remodelling process, computed tomography (CT) studies showed that excessive peri-atrial and pericardial adipose tissue could play a significant role in the loss of atrial architecture and left atrial dilatation [4,5]. However, this could be the main pathophysiologic process in obese patients but not in those with normal or near-normal weight. It has also been shown that obesity and a higher BMI are associated with a higher recurrence rate of atrial fibrillation (AF) after successful catheter ablation (CA) [6]. The same has been proven for the left atrial volume index (LAVI) [7]. It was also proven that there is a correlation between the LAVI and BMI [8]. However, whether LAVI’s prognostic impact on AF recurrence is BMI-independent remains unclear. The aim of this study was to compare correlations between the LAVI and AF recurrence after a successful CA in obese and non-obese patients and to further stratify whether the LAVI’s prognostic impact is the same across the whole BMI spectrum.

## 2. Methods

We prospectively included 62 patients with paroxysmal AF. All patients underwent radiofrequency CA with pulmonary vein isolation (PVI) as the primary target of the procedure. Sex, age, body height, body weight, body mass index (BMI), and type of antiarrhythmic therapy (AAT) were obtained before the procedure.

Prior to ablation, transoesophageal echocardiography was performed in all patients to rule out the presence of thrombus in the LA. The ablation procedure was performed under conscious sedation. Under fluoroscopic guidance, a transseptal puncture was performed. A detailed bipolar voltage map of the left atrium (LA) was then constructed using 20-polar catheter (Pentaray; Biosense-Webster, Irvine, CA, USA). An automated 3D mapping system (Carto, Biosense Webster, Irvine, CA, USA) was used in all patients. We used standard respiratory gating to minimise respiratory movement bias. Prior to ablation, contact force calibration was performed. Endocardial contact was ensured mainly by local electrogram and contact force measurements. Ablation was performed with a 3.5 mm irrigated-tipped catheter (SmartTouch Thermocool, Biosense Webster, Irvine, CA, USA). PVI was achieved with wide antral circumferential ablation. The isolation of the ablated region was then confirmed with entrance and exit block pacing manoeuvres. Direct current cardioversion to restore sinus rhythm was also performed after the successful procedure if patients were still in AF. Mapping and CA were performed by a single operator.

Standard transthoracic two-dimensional echocardiography was performed one day after CA to obtain a standard recording of cardiac function and morphology (Vivid E95, General Electric Vingmed, Milwaukee, WI, USA). Standard two-dimensional and Doppler measurements were obtained according to the current recommendations [9].

### 2.1. Follow-Up

All patients had a 12-lead ECG (25 mm/s, 10 mm/mV) recorded at their follow-up visit 6 months after CA to evaluate their basic rhythm. Patients were also instructed to visit an outpatient clinic earlier in case of symptoms suggesting AF recurrence. We excluded any observed AF during the blanking period: within 3 months after the procedure. In patients where AF was documented with Holter monitoring, this observation was also regarded as AF recurrence. The endpoint of our study was to estimate the AF recurrence rate diagnosed by ECG. Recurrence was defined as documented AF within the first 6 months of the follow-up period.

### 2.2. Statistical Analysis

Obtained data were analysed using SPSS version 26 (SPSS Inc., Chicago, IL, USA). We used Student’s *t*-test for evaluating differences between continuous variables and the chi-square test for the analysis of categorical variables. Continuous data are given as mean ± standard deviation (SD) and categorical variables are expressed as absolute values and percentages. A simple logistic regression method was used to assess the association between the LAVI and AF recurrence. The significance of other potential confounders was adjusted via multivariate logistic regression and the enter method.

## 3. Results

**Figure 1:**
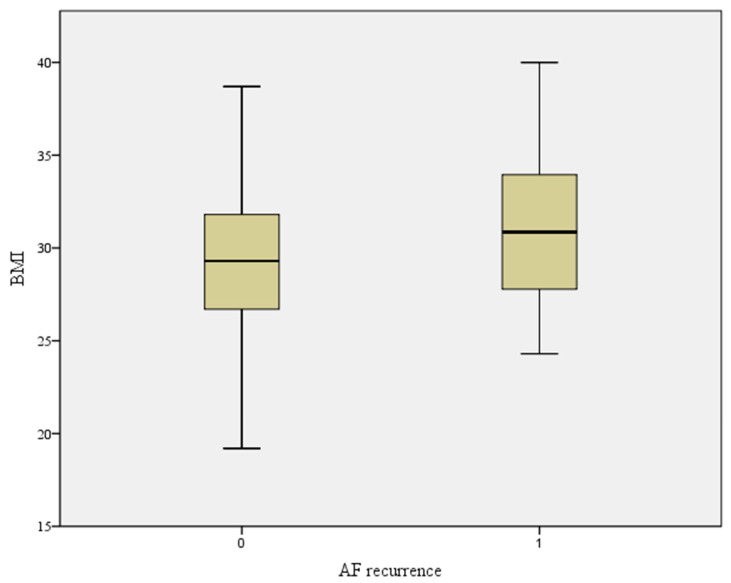
BMI according to AF recurrence. Legend (Figure 1): BMI—body mass index, AF—atrial fibrillation; *p*—0.1.

The patients’ data are displayed in Table 1. We observed AF recurrence in 27% of patients after 6 months. The mean BMI in our cohort was 29.65 ± 5.08 kg/cm^2^ and the mean LAVI was 38.04 ± 11.38 mL/m^2^. BMI was also elevated in AF recurrence patients, but not to the point of statistical significance (Figure 1). We further divided patients into two groups according to BMI (≥30 and <30). We found no statistically significant differences in age, sex, left ventricular ejection fraction, and comorbidities between both groups (Table 2). The LAVI was similar in both groups. There was a slightly higher percentage of AF recurrences in the obese group; however, the difference was not statistically significant (Table 2). We found the LAVI to be a significant predictor of AF recurrence in all patients. When we further divided patients according to BMI, we found the LAVI to be significantly associated with the AF recurrence rate only in obese patients (BMI ≥ 30) and not in normal-weight or overweight patients (BMI < 30) (Table 3). The significance of the LAVI as an AF recurrence predictor according to BMI was also confirmed in a multivariate model in both groups (adjusted for the confounding effects of hypertension, diabetes, left ventricular ejection fraction, and antiarrhythmic therapy) (Table 3).

## 4. Discussion

Previous studies have demonstrated an association between left atrial (LA) size and the incidence of new-onset AF, as well as AF recurrence, after CA [3,6,7]. However, it is still controversial whether this is due to the enlargement of the LA per se or a consequence of the accompanying risk factors, such as obesity and its metabolic derangements. In a recent study, a CT-derived cut-off value for the LAVI of 51.99 mL/m^2^ was proposed as a prognostic marker for AF recurrence after CA, while the impact of BMI and other measures of obesity was not studied [3]. In our study, the LAVI was a predictor of AF recurrence only in obese patients, while no association of the LAVI with AF recurrence was demonstrated in normal-weight and overweight patients.

Obesity was proven to be an independent risk factor for the incidence of AF, with a 10–30% higher risk of AF for every 5 kg/m^2^ increase in BMI [10,11,12]. According to the reported estimations, it already accounts for almost one-fifth of AF cases [13,14]. Interestingly, Pranata et al. demonstrated a non-linear relationship of BMI with AF recurrence after CA, with a steeper curve in those with a BMI >30–35 [6]. Besides hemodynamic stress due to persistent volume overload, obesity was found to increase proinflammatory cytokines, induce insulin resistance, alter metabolic pathways, and induce gene expression profiles associated with cardiac hypertrophy, which result in subsequent electrophysiological, mechanical, and structural LA remodelling [12,15,16,17,18]. Obesity is also commonly associated with other risk factors for LA remodelling and AF, such as arterial hypertension, type 2 diabetes mellitus, and obstructive sleep apnea [19,20]. In obese patients, compared to non-obese patients, a lower global LA longitudinal strain, revealing LA mechanical dysfunction, was also demonstrated [21].

Besides BMI, additional clinical measures, such as waist circumference and waist-to-hip ratio, have been suggested to define regional body fat distribution more precisely and assess visceral fat. A correlation of the BMI and waist-to-hip ratio with visceral adiposity was reported, though it was influenced by gender and race [22,23,24,25]. Extensive evidence has demonstrated the more unfavourable metabolic and cardiovascular effects of visceral compared to subcutaneous fat, mediated by its endocrine proinflammatory and immunological mechanisms [26]. Most studies have evaluated the extra-thoracic part of visceral fat, especially its intra-abdominal distribution, and most of them have confirmed its association with an adverse metabolic phenotype and an enhanced cardiovascular risk [27,28]. In recent years, epicardial adipose tissue (EAT) has been increasingly advocated for as a critical part of visceral fat compartment, associated with LA size and function [5,22,29]. EAT promotes AF by direct fat infiltration of the underlying atrial myocardium, increased oxidative stress, local autonomic dysfunction, accelerated interstitial atrial fibrosis, and subsequent conduction slowing and heterogeneity [28,30,31,32]. EAT and associated inflammatory cytokines were linked with the incidence, severity, and recurrence of AF [33,34].

As noted in a study by van Rosendael et al., the incidence of paroxysmal AF was the highest in patients with a large amount of EAT, while increased LA size predicted AF persistency [5]. Similarly, a recently published meta-analysis confirmed the predictive role of EAT for AF recurrence after CA [35]. Based on these results, an increased EAT in normal-sized LAs might reflect an early LA disease, followed by LA enlargement and the transition from paroxysmal to persistent/permanent AF. Adding our findings to the recent data, one can expect the most frequent AF recurrences in obese patients with a large amount of EAT and subsequent LA enlargement, which warrants further confirmation.

According to our results, LA enlargement unrelated to obesity might have a lower propensity for AF recurrence and the mechanisms behind this are crucial. In non-obese patients, LA enlargement is possibly merely a consequence of prolonged hemodynamic stress due to heterogeneous conditions with LA pressure or volume overload, which can also be aggravated by atrial stand-still during AF episodes. On the other hand, genetic and innervational factors promoting LA enlargement and AF have also been identified [36,37,38]. However, LA enlargement in obese patients seems to carry a higher propensity for AF compared to in non-obese patients, which highlights the additional proarrhythmogenic impact of local and systemic inflammatory conditions mediated by obesity. It is possible that an enlarged left atrium in obese patients reflects different and more aggravating pathophysiological circumstances and processes than in non-obese patients. Due to the inflammatory and direct mechanical impacts of adipose tissue and the additional deranged atrial position in obese patients, it is plausible that there are vast microscopic and cellular changes taking place. Even though the LAVI is similarly enlarged, as in non-obese patients, their atrial micro-architectonics is probably significantly damaged, thus leading to a higher percentage of destructed atrial tissue and hence a higher probability of arrhythmic foci outside of the pulmonary veins and respective antral regions. Further studies that could show more specific differences in atrial tissue with respect to adipose tissue and BMI are needed to prove this hypothesis.

Our results are hypothesis-generating and should be interpreted in the context of certain limitations. In our patients, visceral fat was not specifically evaluated, and our results are based only on BMI. However, BMI is the most frequently used anthropometric measure in clinical routine and its results are easily applied in everyday clinical practise. Since BMI was found to correlate with the amount of visceral fat to a certain degree, we believe that adding more specific measures of visceral fat would not significantly change our results [22]. Among other potential limitations we must mention the lack of data regarding specific antiarrhythmic therapy after the procedure; however, all patients were treated following the current guidelines and there were no major differences in the percentage of prescribed therapy between both groups. We also did not measure the time between the ablation procedure and the registered AF, which would give us further insight in AF recurrence and possible correlations with BMI; however, we postulated that a 3-month window between the blanking period and final observation time would be too short for any meaningful conclusions. Although the number of included patients was limited, all interventional procedures were performed by the same skilled electrophysiologist and echocardiographic examinations were performed by the same experienced echocardiographer on the same ultrasound machine. Thereby, we completely avoided potential interobserver procedural and measurement variability issues. Finally, we also did not perform a 24 h Holter routinely in all patients; however, due to the known low detection value of this method and short observation window, we concluded that symptom-driven diagnostical procedures (Holter or ECG) and clinical presentation with a 12-lead ECG would suffice for the registration of follow-up data [1].

## 5. Conclusions

According to our results, the LAVI tends to be a significant predictor of AF recurrence after CA in obese patients, but not in normal-weight or overweight patients. This would suggest different mechanisms of AF in patients with normal weight or slightly over-weighted patients in comparison to obese patients. Further studies are needed in this regard.

## Figures and Tables

**Table 1:** Patient characteristics.

Parameter	Mean and Standard Deviation
Age (years)	61.52 ± 9.87
Body height (cm)	175.00 ± 9.43
Body weight (kg)	92.13 ± 15.58
LAVI (mL/m^2^)	38.04 ± 11.38
BMI (kg/m^2^)	29.65 ± 5.08

Legend (Table 1): LAVI—left atrial volume index; BMI—body mass index.

**Table 2:** Comparison of patients stratified according to BMI.

	BMI ≥ 30 kg/cm^2^	BMI < 30 kg/cm^2^	*p* Value
Number of patients	27	35	
Age (years)	61.3 ± 11.5	61.7 ± 7.3	0.9
Sex (female)	11(40.7%)	6(17.1%)	0.06
LAVI (mL/m^2^)	38.2 ± 13.2	37.8 ± 8.8	0.9
AF recurrence after 6 months (%)	9(33.3%)	8(22.9%)	0.4
Antiarrhythmic treatment after CA	26(89.7%)	32(84.2%)	0.7
Left ventricular ejection fraction (%)	58.7 ± 2.5	59.3 ± 2.8	0.4
Hypertension	18(66.6%)	19(54.3%)	0.5
Diabetes	1(4.0%)	2(5.7%)	1.0

Legend (Table 2): LAVI—left atrial volume index; AF—atrial fibrillation; BMI—body mass index; CA—catheter ablation.

**Table 3:** LAVI as a predictor of AF recurrence after catheter ablation in both groups.

	Number of Patients	Odds Ratio for AF Recurrence	Confidence Interval	*p* Value
LAVI (BMI < 30 kg/cm^2^)—unadjusted	35	1.08	0.87–1.17	0.1
LAVI (BMI < 30 kg/cm^2^)—adjusted *	35	1.07	0.97–1.17	0.16
LAVI (BMI ≥ 30 kg/cm^2^)—unadjusted	27	1.29	1.07–1.54	0.007
LAVI (BMI ≥ 30 kg/cm^2^)—adjusted *	27	1.35	1.02–1.78	0.03

Legend (Table 3): LAVI—left atrial volume index; AF—atrial fibrillation; BMI—body mass index; * Adjustment for confounding effects of hypertension, diabetes, left ventricular ejection fraction, beta blockers, and antiarrhythmic therapy.

## Data Availability

Authors have the access to all research data.
